# When Testing Went Quiet, the Sewers Kept Talking: A Five-Year SARS-CoV-2 Wastewater Surveillance from Munich

**DOI:** 10.3390/microorganisms14102253

**Published:** 2026-10-04

**Authors:** Jasmin Javanmardi, Jessica Neusser, Raquel Rubio-Acero, Sebastian Kachel, Karina Wallrafen-Sam, Jessica Hintze, Ivana Paunovic, Irene Charlotte Schoof, Thomas Kletke, Maximilian Münchhoff, Andreas Osterman, Caroline Herr, Martin Hoch, Merle M. Böhmer, Alexander Klaus, André Klima, Elisabeth Waldeck, Michael Hoelscher, Christof Geldmacher, Roman Wölfel, Jan Hasenauer, Peter Braun, Andreas Wieser

**Affiliations:** 1 Institute of Infectious Diseases and Tropical Medicine, LMU University Hospital, LMU Medizin, Ludwig-Maximilians Universität München, 80802 Munich, Germany; jasmin.javanmardi@med.uni-muenchen.de (J.J.); raquel.rubio@med.uni-muenchen.de (R.R.-A.); jessica.hintze@uni-greifswald.de (J.H.); irenecharlotte.schoof@med.uni-muenchen.de (I.C.S.); hoelscher@lrz.uni-muenchen.de (M.H.); geldmacher@lrz.uni-muenchen.de (C.G.); peter.braun@itmp.fraunhofer.de (P.B.); 2 Bavarian Health and Food Safety Authority, 80339 Munich, Germany; jessica.neusser@lgl.bayern.de (J.N.); caroline.herr@lgl.bayern.de (C.H.); martin.hoch@lgl.bayern.de (M.H.); merle.boehmer@lgl.bayern.de (M.M.B.); 3 Immunology, Infection and Pandemic Research, Fraunhofer Institute for Translational Medicine and Pharmacology ITMP, 80799 Munich, Germany; sebastian.kachel@itmp.fraunhofer.de (S.K.); ivana.paunovic@itmp.fraunhofer.de (I.P.); 4 Life & Medical Sciences Institute (LIMES), University of Bonn, 53115 Bonn, Germany; karina.wallrafen@uni-bonn.de (K.W.-S.); jan.hasenauer@uni-bonn.de (J.H.); 5 Bonn Center for Mathematical Life Sciences, University of Bonn, 53115 Bonn, Germany; 6 Munich Metropolitan Sewer Authority (MSE), 81671 Munich, Germany; 7 Max von Pettenkofer Institute of Hygiene and Medical Microbiology, Faculty of Medicine, LMU Munich, 80336 Munich, Germany; muenchhoff@mvp.lmu.de (M.M.);; 8 German Center for Infection Research (DZIF), Partner Site Munich, 80802 Munich, Germany; romanwoelfel@bundeswehr.org; 9 Institute and Outpatient Clinic for Occupational, Social and Environmental Medicine, LMU University Hospital, Ludwig-Maximilians-Universität (LMU) München, 80336 Munich, Germany; 10 Pettenkofer School of Public Health, Chair of Public Health and Health Services Research, Ludwig-Maximilians-Universität (LMU) München, 81377 Munich, Germany; 11 Comprehensive Pneumology Center Munich (CPC-M), Member of the German Center for Lung Research (DZL), 81377 Munich, Germany; 12 Institute of Social Medicine and Health Systems Research, Otto-von-Guericke-University Magdeburg, 39120 Magdeburg, Germany; 13 Public Health Department Munich (GSR), 80335 Munich, Germany; alexander.klaus3@muenchen.de (A.K.); andre.klima@muenchen.de (A.K.); elisabeth.waldeck@muenchen.de (E.W.); 14 Unit Global Health, Helmholtz Zentrum München, German Research Center for Environmental Health, 85764 Munich, Germany; 15 Bundeswehr Institute of Microbiology, 80937 Munich, Germany

**Keywords:** COVID-19, SARS-CoV-2, wastewater, epidemiology, sewage, surveillance, longitudinal, variant of concern, pandemic, clinical cases, Google Trends

## Abstract

Wastewater-based epidemiology (WBE) was introduced early in the COVID-19 pandemic to provide continuous, anonymous, population-level information on SARS-CoV-2 circulation at a time when individual testing was constrained by shortages of reagents, staff, logistics, and funding. It has been difficult to assess whether wastewater data retain their epidemiological value once clinical testing declines, because most published records cover only the first one or two pandemic years. We monitored SARS-CoV-2 in Munich wastewater continuously from April 2020 to April 2025, spanning all lockdown and post-lockdown phases, the vaccination campaigns, and the first post-pandemic years. Samples were collected once or twice weekly as qualified morning grab samples from six residential catchments, and as 24-h composite samples from the wastewater treatment plant (WWTP) Gut Grosslappen. A total of 1100 samples were analyzed by RT-qPCR or droplet digital PCR (ddPCR), and compared with official notification data for the corresponding catchment areas. Weekly viral loads correlated significantly with reported cases at every site (Spearman ρ 0.47 to 0.79), with no consistent lead or lag at weekly resolution. Correlations were higher for the residential catchments (ρ 0.62 to 0.79) than for the WWTP (ρ 0.47 and 0.58), although this difference did not persist when the comparison was restricted to an interval in which both site types were sampled concurrently. The ratio of wastewater viral load to reported cases remained near baseline while testing was mandatory, and rose markedly after mandatory testing ended in March 2023. This shows that notification data increasingly underestimated infection numbers while wastewater continued to track circulation. Wastewater surveillance therefore becomes more informative, not less, as clinical surveillance contracts.

## 1. Introduction

The COVID-19 pandemic set the world in an extraordinary state of emergency for more than three years [1]. With the identification of the first clinical case of a SARS-CoV-2-infected individual in Wuhan, Hubei province, China, healthcare providers and authorities prioritized the expeditious identification and isolation of persons infected with the virus. Subjects who met the case definition, and their close contacts, were screened using reverse transcription polymerase chain reaction (RT-PCR) techniques [2]. The challenges experienced during this period included monitoring the spread of SARS-CoV-2 among the population, the development and implementation of testing strategies and capacities, the management of hospitalizations, and the identification of asymptomatic cases. These challenges had a significant impact on healthcare and educational units [3].

As the pandemic progressed, limitations of the testing strategy employed in numerous countries became apparent. In Germany, for instance, diagnostic PCR capacities were limited in early 2020, with testing primarily focused on symptomatic individuals [4]. Rapid diagnostic antigen tests became available by late 2020 for use in medical care and screening settings, and in early-to-mid 2021 for general public and home use. However, these tests demonstrated lower or variable sensitivity [5,6,7]. In the latter third of the pandemic, a considerable expansion in diagnostic capacities could be achieved. However, the population’s willingness to be tested declined markedly, owing to the discomfort associated with deep nasal swabs, quarantine regulations, and the availability of vaccines offering effective personal protection against severe symptoms and death [8,9]. A study has demonstrated that the efficacy of a given testing strategy is contingent upon two primary factors: the frequency of testing and the swiftness of reporting results [10]. The high proportion of untested, oligo- or asymptomatic individuals resulted in severe under-reporting of SARS-CoV-2 infections. Yet decisions of health authorities depend on solid and trustworthy data. In the past, mandates for masking or even lockdowns were based on data from the compulsory reporting of new SARS-CoV-2 infections and the number of hospital beds occupied by COVID-19 patients, or on COVID-19-related deaths as key indicators of disease burden [11,12].

Early during the COVID-19 pandemic, studies revealed the presence of SARS-CoV-2 RNA in stool samples of infected individuals, both those exhibiting symptoms and those with oligo-/asymptomatic infections [13]. Prior to the pandemic, SARS-CoV had already been documented in wastewater [14]. Consequently, efforts to ascertain the viral load of SARS-CoV-2 in sewage commenced promptly in 2020. It rapidly became evident that wastewater constituted an intriguing medium for the detection of SARS-CoV-2, with the viral RNA exhibiting sufficient stability to facilitate the implementation of RT-PCR-based methodologies for its detection and quantification [15,16,17].

Subsequent investigations have indicated a notable correlation between the concentration of SARS-CoV-2 RNA in wastewater samples and the actual prevalence or notification numbers of COVID-19 in a population [18,19]. This suggests that municipal wastewater viral loads, or information on circulating variants, can offer spatiotemporal information on the infection activity of SARS-CoV-2 in populations, thereby assisting public health authorities in making evidence-based decisions for outbreak mitigation [20,21]. Most SARS-CoV-2 variants of concern are distinguished by mutations in the gene encoding the spike (S) protein, which mediates host–cell entry and is the principal target of neutralizing antibodies. Because these substitutions and deletions cluster in the S gene, they also underpin routine variant detection: several widely used multiplex RT-PCR assays target the S gene alongside ORF1ab and N, and failure to amplify the S target while the remaining targets stay positive has served as a rapid proxy for variant circulation, including in settings without access to genomic sequencing [22]. Moreover, wastewater-based epidemiology (WBE) represents a cost-effective alternative to individual clinical screening and traditional reporting systems in both resource-limited and high-income settings [23]. By substantially reducing the number of diagnostic analyses required, it yields robust community-level surveillance data at lower logistical and financial cost.

A limited number of research groups have collected wastewater data continuously during and after the pandemic. Many groups have focused on a shorter period of three to 14 months, in the first and/or second year (2020/2021) of the pandemic [24,25,26,27,28]. To the best of our knowledge, no study has covered SARS-CoV-2 surveillance in wastewater continuously from the beginning of the pandemic for five consecutive years, longitudinally comparing the data with the number of notified cases throughout lockdown, inter-lockdown, and post-pandemic phases. Only recently, a more comprehensive four-year study in Miami, US, compared SARS-CoV-2 levels at three wastewater treatment plants (WWTPs) against clinical metrics [29]. Our five-year dataset from Munich expands upon this by capturing the complete transition from emergence to epidemic phase into the long-term endemic phase up to April 2025. This was done in a completely different setting in Europe, with distinct temperature and climatic conditions. Furthermore, in our study we provide data from six smaller catchment areas as well as the main WWTP in Munich.

In our previous report from 2021 [30], we demonstrated WBS of SARS-CoV-2 over the initial year of the pandemic, visualizing the two infection waves of 2020/2021 and the influx of the Alpha variant into the Munich area. This variant was detected in wastewater before it appeared in the sequencing results of swabs sent to the health authorities. Here we extend that work to five consecutive years of wastewater monitoring in Munich, from April 2020 to April 2025, covering all phases of the pandemic: the lockdowns, the post-lockdown period, the vaccination campaigns, and the successive changes to the national testing strategy. Sampling was carried out once or twice per week as qualified morning grab samples at six residential catchments across Munich, in Bavaria, southern Germany. In addition, 24-h (24 h) composite samples were collected regularly at the main wastewater treatment plant, Gut Grosslappen, in the north of the city. The novelty of this work lies in the longitudinal, spatially resolved dataset, which spans multiple shifts in public health testing and surveillance regimes. Furthermore, this study shows how the epidemiological value of wastewater surveillance changes as clinical surveillance evolves from intensive pandemic-era testing to the substantially reduced testing of the post-pandemic period.

Using this dataset, we address one primary question: how well SARS-CoV-2 wastewater viral loads track reported case numbers over time, and whether this relationship changes as clinical testing declines. Closely linked to this, we examine whether the viral-load-to-case ratio reveals underreporting once mandatory testing ends. Four secondary, exploratory analyses make further use of the same longitudinal record: whether lockdowns leave a measurable signature in the wastewater signal; how the timing of variant-specific peaks maps onto the succession of dominant lineages; whether public search interest, measured through Google Trends, carries any signal comparable to the wastewater data; and whether periods of large mass gatherings coincide with changes in wastewater viral loads. These secondary analyses are hypothesis-generating rather than confirmatory and are reported without claims of causal inference. Together, these analyses assess whether long-term wastewater surveillance provides valuable additional insights into community infection dynamics even as clinical testing wanes, which is critical to understanding its potential role in supporting evidence-based public health decisions.

## 2. Materials and Methods

### 2.1. Wastewater Sampling

**Figure 1:**
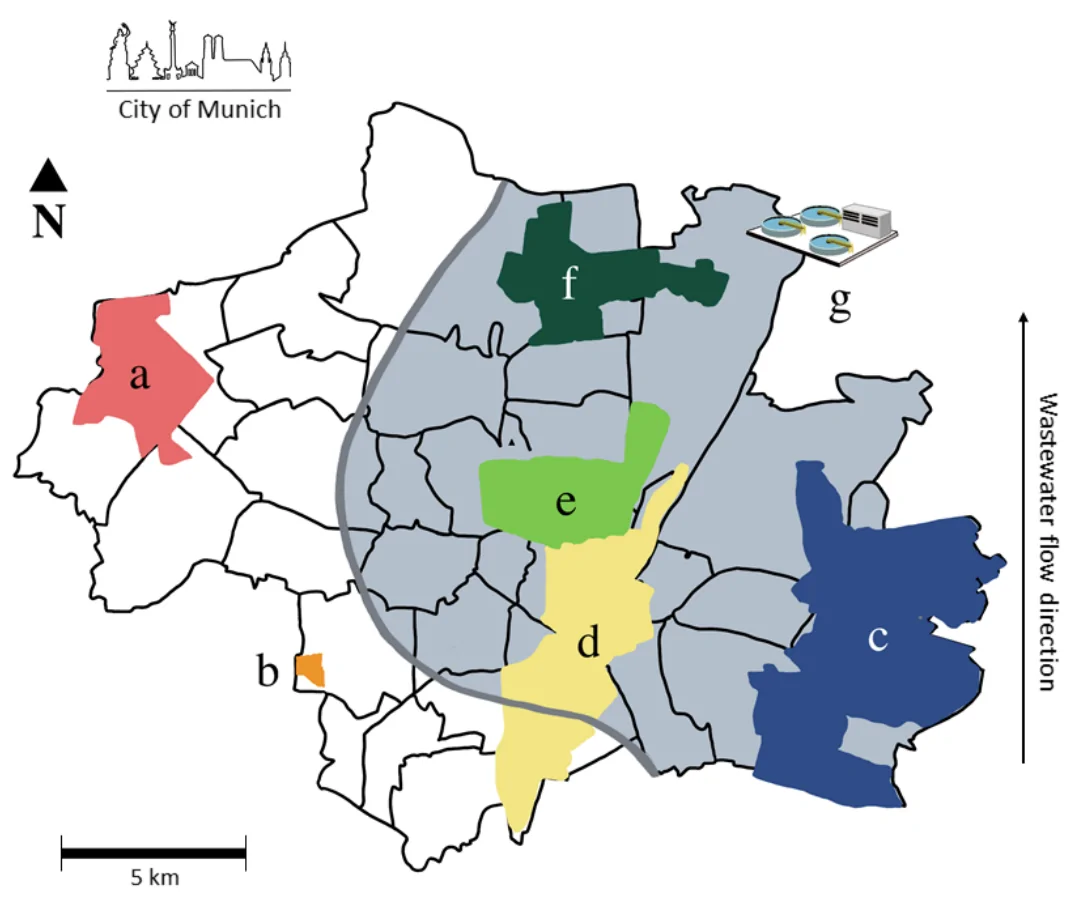
Map of the city of Munich annotated with sampled catchment areas. (a) Langwieder Bach: red, (b) Grosshadern: orange, (c) Savitstr.: blue, (d) Gysslinger Becken: yellow, (e) Schenkendorfstr.: light green, (f) Schmidbartlanger: dark green, and (g) WWTP Gut Grosslappen: grey. WW flows from south to north. North indicated by an arrow. Black lines within city map indicate different neighborhoods. Panel letters (a)–(f) denote the six residential catchments and are used in this order throughout all figures and tables. Letters beyond (f) denote additional panels within the respective figure only and do not identify sampling sites.

Wastewater sampling, transportation, processing and storage were conducted as described previously [30] for the period April 2020–April 2025 in Munich, Germany. During the study, the number of sampling sites was reduced and their locations were changed to simplify sample logistics (see Section 3.1 for details). Sampling sites are listed in Table 1 and Figure 1. The six residential catchments are designated (a) to (f), and the treatment plant is designated WWTP. The spot sampling time was chosen to reflect the morning flush surge. Frequency of sampling was increased from once to twice per week in mid-November 2021 as recommended by the European Union (EU) [31].

### 2.2. Virus Concentration and Nucleic Acid Extraction

The virus in the wastewater (WW) was concentrated by centrifugation as previously established [30]. This protocol was used for nucleic acid extraction until mid-September 2022. Following concentration by centrifugation, automated nucleic acid extraction was performed using TANBead Maelstrom 4810 (Taiwan Advanced Nanotech Inc., Taipei, Taiwan) with the TANBead Optipure Viral Auto Tube M665S46 Isolation Kit (LabConsulting GmbH, Vienna, Austria). A comparative analysis was conducted on the performance of the automated extractor and the manual kit, which revealed that they were generally similar, exhibiting approximately one C_t_-value difference. To enhance the efficiency and comparability of results, the automated protocol was utilized from mid-September 2022 onwards.

As a brief summary, 50 mL of raw WW was centrifuged to remove debris and bacteria, and subsequently 38 mL of supernatant was centrifuged in an ultracentrifugation step. The pellets were resuspended in 300 µL nuclease-free water (VWR, 436912C, Darmstadt, Germany). The resuspended pellet was transferred to the extraction tube, adding 10 µL of proteinase K. The extraction program “667 rapid”, which had been pre-programmed in the “Maelstrom 4810” instrument, was utilized in this study. Whole nucleic acids were eluted in 60 µL of elution buffer. Samples were stored at 4 °C for a maximum of five days, or −80 °C for long-term storage before analysis.

### 2.3. RT-qPCR and ddPCR (BioRAD)

Until November 2022, samples were analyzed by RT-qPCR, as previously described [30]. In summary, a primer/probe combination as outlined in the CDC protocol was utilized. Amplification was conducted with a one-step QuantiNova Multiplex RT-PCR kit on a LightCycler 480 II (Roche, Switzerland). Standard curves and quantifications were maintained in accordance with previously published protocols [30]. Viral loads were calculated as copy numbers per liter of sewage.

Beginning in November 2022, samples of the WWTP Gut Grosslappen were analyzed by BioRAD (Feldkirchen-Westerham, Germany) droplet digital PCR (ddPCR). This methodological upgrade significantly enhanced diagnostic sensitivity. In WBE, this increased sensitivity is critical due to environmental confounding factors, such as heavy precipitation events, that can dilute sample concentrations and push target genomic copies below the detection limits of standard RT-qPCR, potentially masking the early acceleration phases of community infection waves [32]. The SARS-CoV-2 RNA targets ORF-1a and E-gene were absolutely quantified in purified wastewater samples [33]. The reaction mix contained 5 µL template RNA, 5 µL One-Step RT-ddPCR advanced supermix (final concentration 1×), 2 µL reverse transcriptase (20 U/µL final concentration), 1 µL E-gene primer/probe mix, 1 µL ORF1a primer/probe mix (both final concentration: 900 nM primers, 250 nM probe), 1 μL of dithiothreitol (DTT) (final concentration 15 nM), and 5 µL nuclease-free water. Droplets were generated with a QX200 ddPCR droplet generator. The information for primers is listed in Table 2. RT-qPCR was run on a Master cycler Pro (2 °C/s ramp) (Eppendorf, Wesseling-Berzdorf, Germany) under the following thermal conditions: 50 °C for 60 min (RT), 95 °C for 10 min (enzyme activation), 40 cycles of 94 °C for 30 s (denaturation) and 58 °C for 1 min (annealing/extension), 98 °C for 10 min (final inactivation) and a cool-down to 4 °C. After overnight incubation at 4 °C on the cycler, droplets were analyzed using QX100 droplet reader and QuantaSoft Pro software (version 2.1.0.25, BioRAD Laboratories, Munich, Germany).

Since the E gene is also present in subgenomic RNAs, ORF1a was selected for analysis, as it is confined to the genomic RNA and therefore more representative of genome copy numbers [34]. The S gene was not targeted due to its high evolutionary rate and frequent mutations across variants.

**Table 2:** Information about primers used in droplet digital PCR (BioRAD).

Target	Primer and Sequence	Reference
E-gene (FAM)	E Sarbeco F1 ACA GGT ACG TTA ATA GTT AAT AGC GT E Sarbeco R2 ATA TTG CAG CAG TAC GCA CAC A E Sarbeco P1 FAM-ACA CTA GCC ATC CTT ACT GCG CTT CG-BHQ1	[2]
ORF 1a (HEX)	Noblis.12 F ACG GCA GTG AGG ACA ATC AG Noblis.12 R CTG CAA CAC CTC CTC CAT GT Noblis.12 probe HEX-CCA ACA GTG GTT GTT AAT GCA GCC A-BHQ1	[35]

### 2.4. Data Normalization with PMMoV

Owing to relatively consistent pepper consumption across a human population in a certain region, Pepper Mild Mottle Virus (PMMoV) enters the WW stream at a stable and predictable concentration, allowing deviations from this baseline to be attributed to dilution from rainfall, snowmelt, or sudden household or industrial water inputs [36]. Thus, PMMoV was used for normalizing data, determined for each catchment area. This applies to all samples analyzed with ddPCR, as this method was established after November 2022. For all samples before November 2022, viral loads of SARS-CoV-2 were normalized retrospectively with PMMoV. Thus, all presented data were normalized with the fecal indicator.

The quantification of PMMoV was conducted by RT-qPCR as described by the manufacturer, using the kit “GoTaq Enviro PMMoV Quant Kit, Quasar 670” (Promega GmbH, Walldorf, Germany). In summary, 5 µL of a 1:10 sample dilution was added to 10 µL of Master Mix, 1 µL of primer/probe mix, 3.6 µL of nuclease-free water, and 0.4 µL of enzyme mix. The thermal cycling protocol, as described by the manufacturer, in conjunction with the fluorophore Quasar 670/Cy5, was utilized for a total of 40 cycles.

### 2.5. Reported Notification Data of COVID-19 Cases

According to the German Infection Protection Act (IfSG) § 6 and 7, SARS-CoV-2 detections in clinical samples and infections are reported to the local health authorities. This generates a precise dataset of laboratory-confirmed cases, including the usual whereabouts of each subject. Intersecting this data with the geofences of each catchment area, all subjects who tested positive via rapid test and/or PCR test in a certain timeframe and area can be correlated with the respective wastewater concentrations of SARS-CoV-2.

### 2.6. Timeline of Lockdowns in the Federal State of Bavaria

Three successive lockdowns were introduced by the Bavarian state government to mitigate uncontrolled infection dynamics. Table 3 shows the exact dates for each lockdown, inter-lockdown, and post-lockdown period.

### 2.7. Data and Statistical Analysis

**Figure 2:**
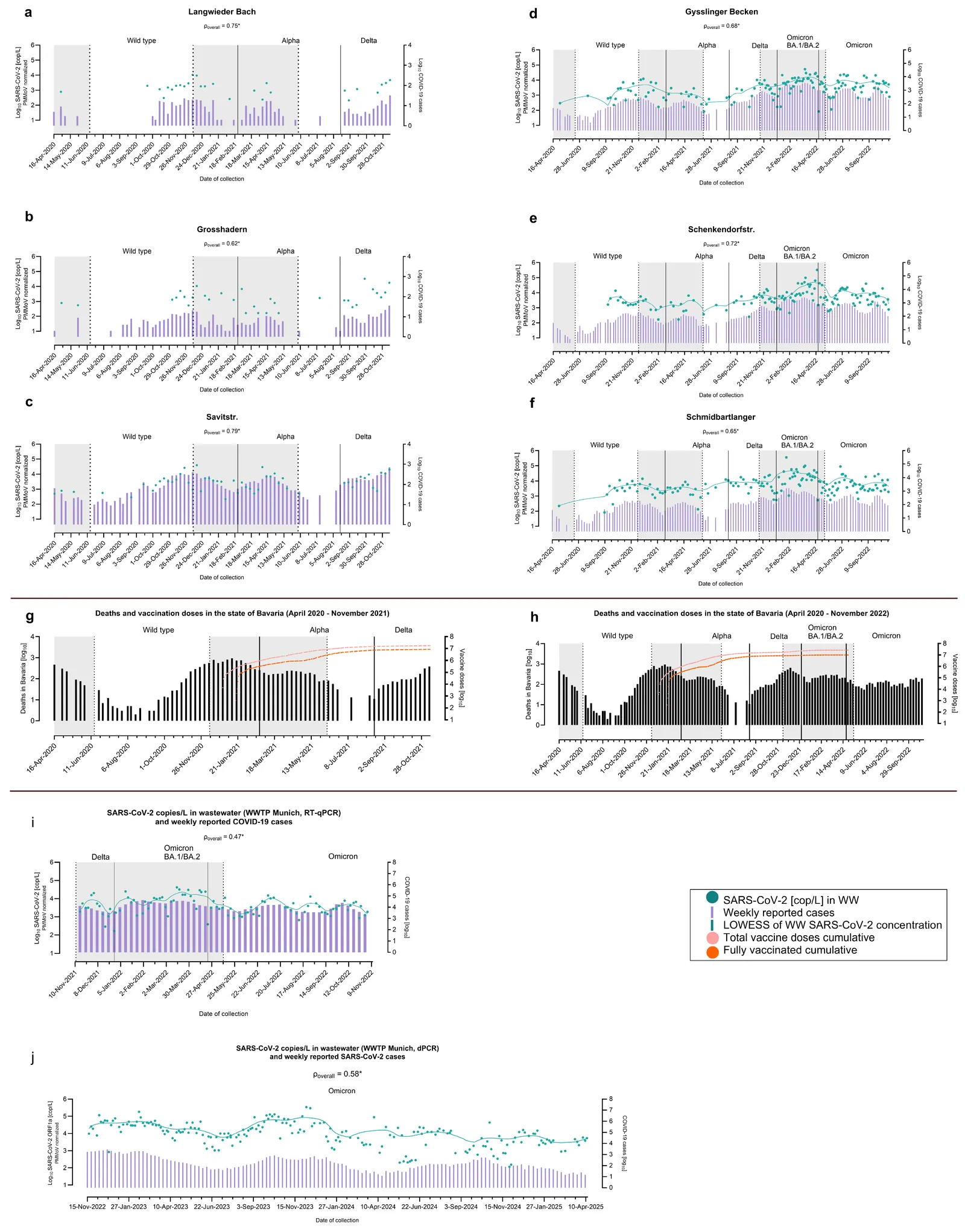
Longitudinal spatio-temporal dynamics of SARS-CoV-2 viral loads across residential wastewater catchment areas in Munich. RT-qPCR and ddPCR monitoring of log_10_ wastewater viral loads normalized to PMMoV (turquoise dots), and log_10_ weekly reported clinical cases (purple bars) to the local health authority of Munich (GSR) tracked across six residential catchments: (**a**) Langwieder Bach, (**b**) Grosshadern, (**c**) Savitstr., (**d**) Gysslinger Becken, (**e**) Schenkendorfstr., (**f**) Schmidbartlanger, (**i**) WWTP Gut Grosslappen analyzed by RT-qPCR, and (**j**) WWTP Gut Grosslappen analyzed by ddPCR. Sampling time frames span from April 2020 to November 2021 for (**a**–**c**), from April 2020 to November 2022 for (**d**–**f**), November 2021 to November 2022 for (**i**), and November 2022 to April 2025 (**j**). Spearman correlation coefficients (ρ) for each catchment area are added to the respective graph; corresponding *p*-values are listed in Appendix A Table A1, additional ρ correlations in Figure A1. Asterisks indicate significance. Shaded grey backgrounds denote official public health lockdown periods. LOWESS regression trends for wastewater viral dynamics are indicated by the solid turquoise lines in panels (**d**–**f**). Panels (**g**,**h**) show deaths in Germany from April 2020 onwards (black bars) and cumulative vaccination rates for fully vaccinated individuals (orange) and the total vaccine doses (pink) in Bavaria between January 2021 and April 2022 [41]. Panels (**a**–**f**) denote the residential catchments as defined in Figure 1; panels (**g**–**j**) are specific to this figure and do not identify sampling sites.

**Figure 3:**
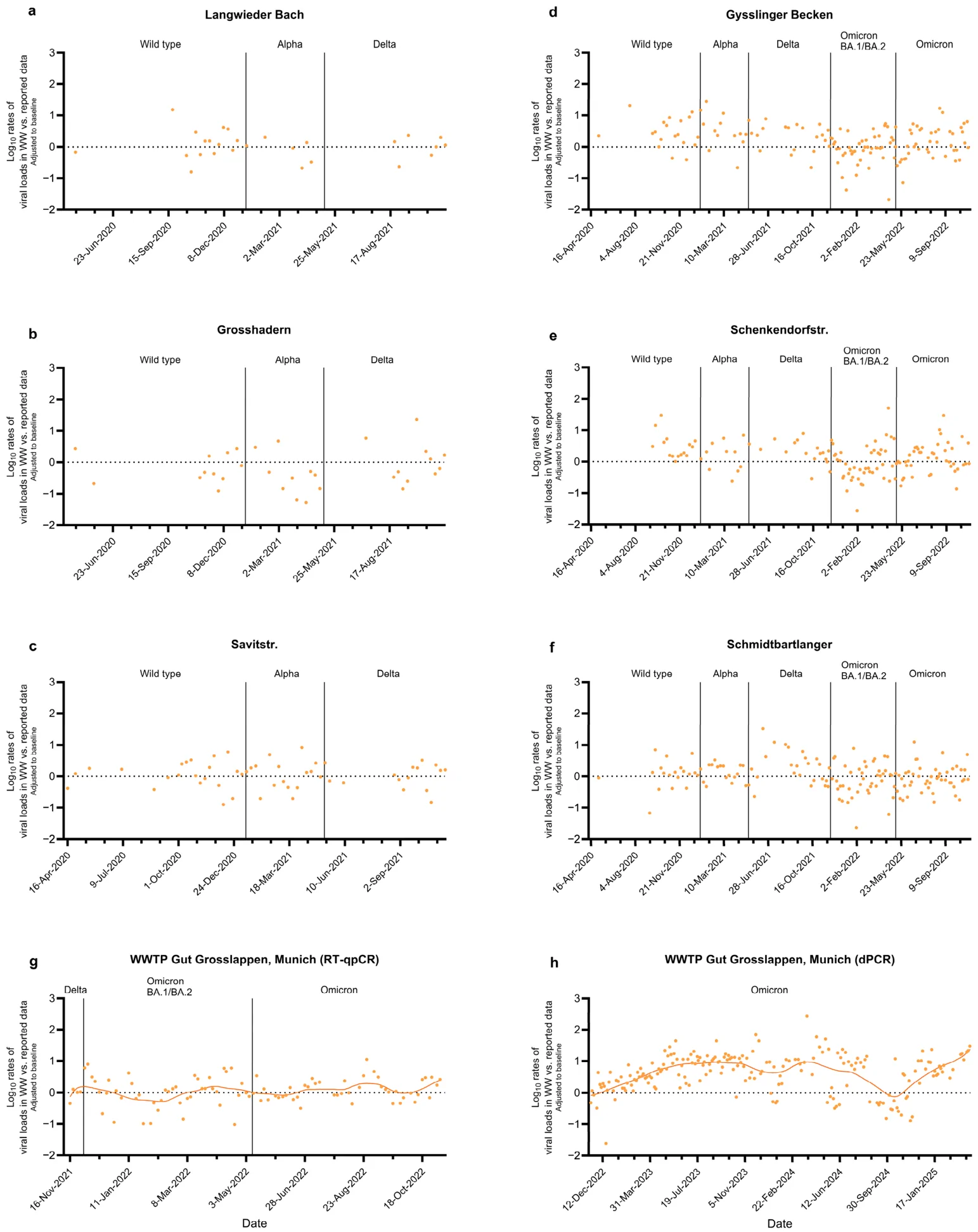
Ratio of wastewater SARS-CoV-2 viral loads to reported case numbers over time under changing testing conditions. For each location, the log_10_ ratio of wastewater viral load to reported COVID-19 cases, referenced to a location-specific reliable-reporting baseline (dotted line at 0), is plotted against sampling date. Each point (orange) represents one sample. Panels (**a**–**f**) show the residential spot-sampling catchments, (**g**) WWTP Gut Grosslappen quantified by RT-qPCR, and (**h**) WWTP Gut Grosslappen quantified by ddPCR. Trendline (orange) was added for the WWTP to visualize the upward trend after the lifting of mandatory testing. Vertical lines divide the periods of the different variants’ dominance. Values near zero indicate agreement between the wastewater and case-based signals. Positive deviations indicate case reporting underascertainment relative to the wastewater signal.

Viral loads over time (Figure 2) and the ratios of WW SARS-CoV-2 loads to clinical cases (Figure 3) were analyzed and visualized with GraphPad Prism (version 10.6.1, (892)). LOWESS (Locally Weighted Scatterplot Smoothing) analysis was conducted with 20 values in the smoothing window and fitted for WW data where applicable. For locations (a–c) LOWESS analysis was not applied due to an insufficient number of data points.

**Figure 4:**
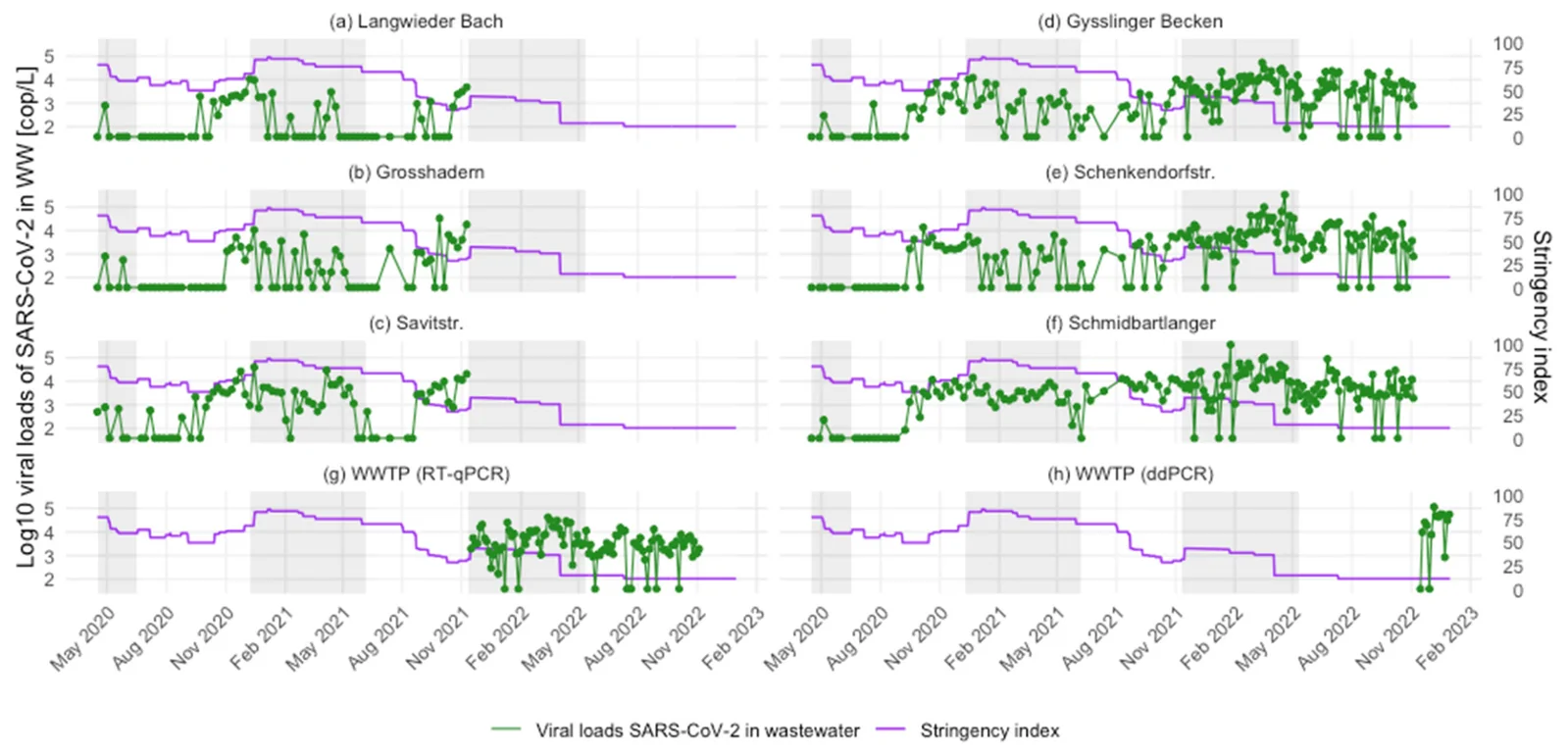
Wastewater SARS-CoV-2 trends and their relationship to lockdown stringency. Time series of wastewater viral load (log_10_, green) for each site overlaid with the national lockdown stringency index (0–100, purple). Shaded bars indicate lockdown periods.

**Figure 5:**
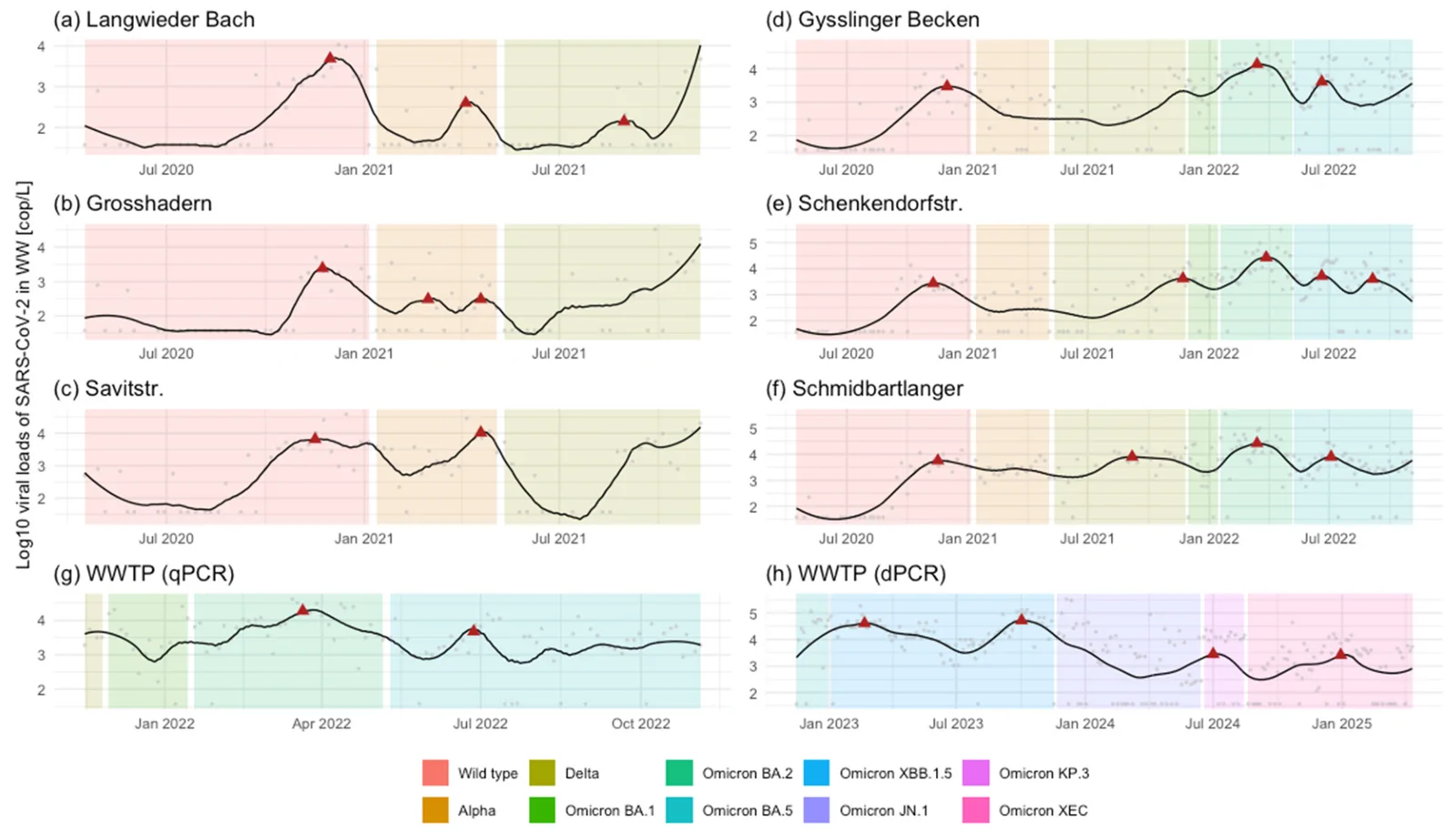
Variant-specific peaks in wastewater SARS-CoV-2 viral load across sampling sites. For each site, daily log_10_ viral loads (points) were smoothed by LOESS (span 0.2, degree 2; black curve) and screened for local maxima using a topographic-prominence filter (minimum prominence 0.3 log_10_, minimum 3-week separation). Detected peaks are marked (red triangles). Background shading indicates the contemporaneously dominant SARS-CoV-2 variant (see legend). Panels covering later dates derive from WWTP ddPCR monitoring, extending variant coverage through XEC at the end of 2024.

**Figure 6:**
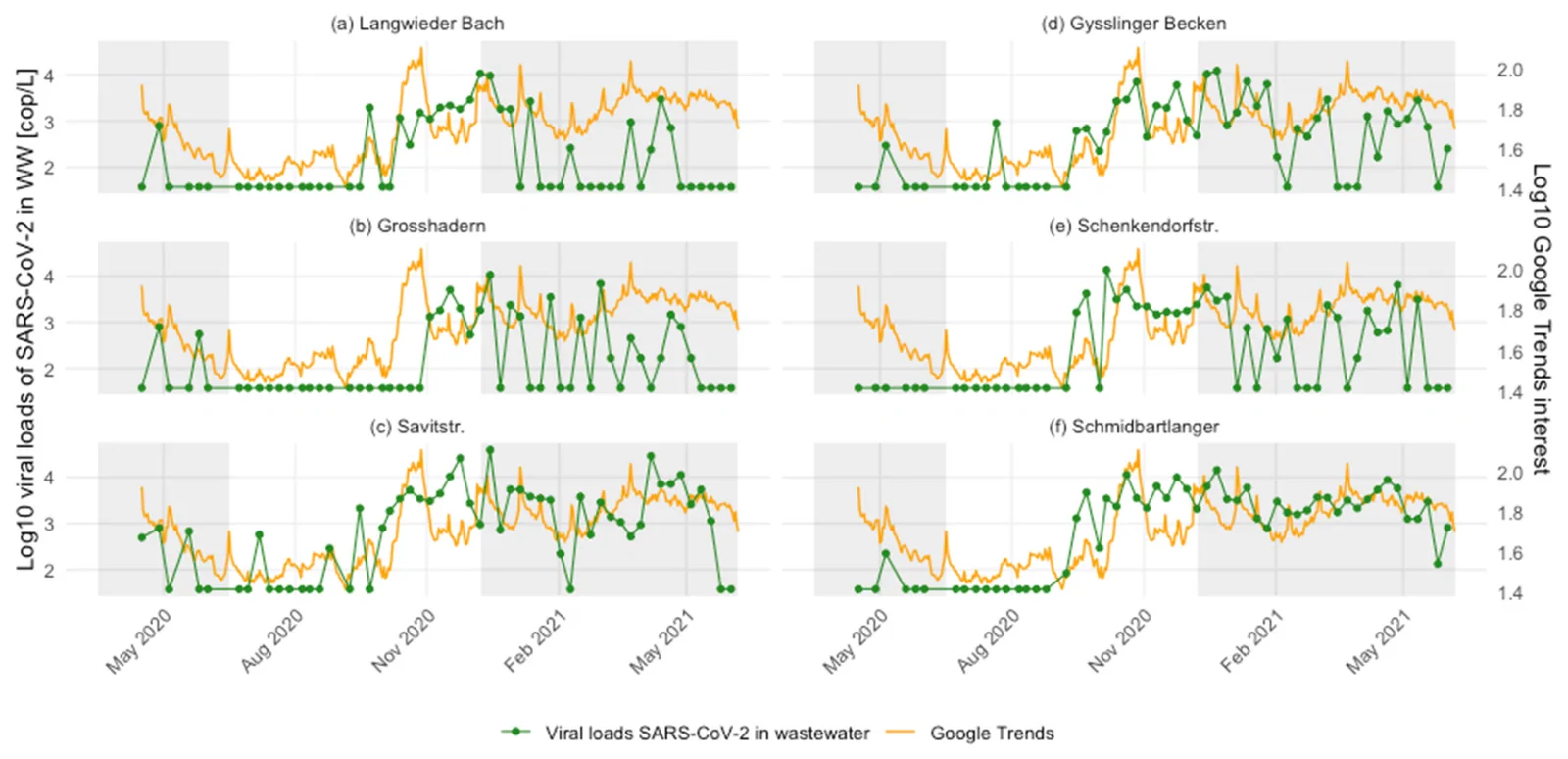
Relationship between wastewater SARS-CoV-2 viral load and Google Trends search interest. For each catchment site (**a**–**f**) and period, wastewater viral load (log_10_, green) is shown together with the daily relative Google Trends search interest for the term “corona” in Bavaria (log_10_-scale, orange), retrieved via the trendecon package. Shaded bars indicate lockdown periods. Spearman lag correlations were evaluated over ±21 days in 3-day steps. A clear positive association, with wastewater leading search interest by 0–3 days, was confined to the first inter-lockdown interval.

All other statistical analyses and visualizations were conducted in R (version 4.4.1) using RStudio (versions 2024.04.2 + 764 and 2026.01.1 + 403), including visualization of WW trends and stringency (Figure 4), variant-specific peaks in WW (Figure 5), and Google Trends data and viral loads in WW (Figure 6). Packages used for analysis were trendecon (v0.3.2), pracma (v2.4.6), and nlme (v3.1-166). LOESS analysis was conducted in R with a span of 0.2 and degree of 2 for variant peak timing.

### 2.8. Details on Statistical Analysis

For the analysis of whether viral loads reflect case numbers, for each of the monitored sites, weekly geometric-mean log_10_ viral loads were correlated to the corresponding weekly log_10_ reported case counts, as levels and as week-over-week growth rates. Spearman correlations were computed overall, stratified by the latest variant reaching 5% prevalence, and by pandemic period; cross-correlations were examined for lags of up to ±4 weeks. To account for temporal autocorrelation in the weekly series, *p*-values were adjusted using Bartlett’s effective sample size formula [37]; since the correlations were rank-based, the autocorrelations were estimated on the ranked series accordingly. BH false-discovery-rate correction was then applied within each stratum (period or variant) and metric combination. See Section 3.2 for results.

For the analysis of viral load dynamics during and after lockdowns, for each site, per-period trends in log_10_ viral loads were estimated as slopes from generalized least-squares (GLS) models incorporating a continuous-time first-order autoregressive (corCAR1) error structure. Only cells with n ≥ 8 observations were interpreted, which excluded the first lockdown, and slopes were subjected to global BH correction. Independently, viral load was Spearman correlated with the national OxCGRT stringency index [38], with *p*-values again adjusted for autocorrelation using Bartlett’s formula and for multiplicity using BH correction. See Section 3.4 for results.

For the analysis of variant-specific peaks in WW, daily log_10_ viral loads were smoothed per site by LOESS (see Section 2.7), and collapsed onto a weekly grid. Screening for peaks was conducted using a topographic-prominence filter with minimum prominence of 0.3 log_10_ and a minimum three-week separation. Data from the Bavarian project BayVOC were used to identify the latest variant reaching 5% prevalence of VOCs [39]. Detected peaks were then mapped. See Section 3.5 for results.

For the comparison of Google trends to viral loads of SARS-CoV-2 in WW, daily relative search interest for the term “corona” in Bavaria was retrieved from Google Trends [40] via the trendecon R package (see Section 2.7) for 16 April 2020 until 11 April 2025, and correlated with per-site viral load using Spearman lag correlations over ±21 days in three-day steps, evaluated separately by site and period. A two-step procedure was applied, where the contemporaneous (lag 0) association was first evaluated with BH correction, and the optimal lag was scanned only for cells with lag-0 |ρ| ≥ 0.5. Given the reduced effective sample sizes, this analysis is treated as exploratory and results are described by the magnitude and direction of the correlation coefficient rather than by significance. See Section 3.6 for results.

Analyses were structured hierarchically. The relationship between wastewater viral loads and reported cases (Section 3.2) and the viral-load-to-case ratio under changing testing conditions (Section 3.3) constitute the primary analyses, addressing the study’s central question. The analyses of lockdown-associated dynamics (Section 3.4), variant-specific peak timing (Section 3.5), search interest (Section 3.6), and mass gatherings (Section 3.7) are secondary and exploratory. They are hypothesis-generating uses of the same longitudinal dataset, are not powered as formal tests, and are reported without claims of causal inference.

Because ddPCR was applied only to WWTP samples from November 2022 onward, while all residential catchments were quantified by RT-qPCR throughout, no time series in this study spans both platforms. All WWTP analyses were therefore performed separately for the RT-qPCR and ddPCR periods, each with its own reference baseline, and no measurements were pooled across platforms.

## 3. Results and Discussion

The primary question addressed in this study is how well SARS-CoV-2 wastewater viral loads track reported case numbers, and whether this relationship changes as clinical testing declines. Section 3.2 and Section 3.3 address this directly, while the remaining subsections present secondary, exploratory analyses that make use of the same five-year record. Over five consecutive years, between April 2020 and April 2025, we collected 1100 samples from seven locations in Munich (Table 1). Six locations were probed with qualified spot samples, and the seventh was a twenty-four-hour (24 h) composite sample of the WWTP Gut Grosslappen inflow (Figure 1). All samples were tested for SARS-CoV-2 genome copy numbers using RT-qPCR or ddPCR. Changes in infection dynamics associated with lockdowns and post-lockdown periods were identified and compared to officially reported clinical data of the respective catchment area.

### 3.1. Decentralized Wastewater Surveillance Visualized over the Period of Five Years

At the beginning of the COVID-19 pandemic in April 2020, sampling was initiated at different locations within Munich. Spot sampling was conducted at catchment areas Langwieder Bach (a), Grosshadern (b), Savitstr. (c), Gysslinger Becken (d), Schenkendorfstr. (e), and Schmidbartlanger (f). The first three locations were sampled until November 2021, and the latter three until November 2022. Initially, sampling was conducted once per week until November 2021, after which it was increased to twice per week. Viral loads were measured by RT-qPCR and subsequently compared to reported cases of the respective residential area as obtained by the Health Authority of Munich (GSR). Sampling of the WWTP was initiated in November 2021 and continued twice weekly until April 2025. Viral loads were measured with RT-qPCR until November 2022 (Figure 2i), followed by ddPCR until April 2025 (Figure 2j), as ddPCR is more sensitive to lower viral loads.

The sampling timelines span different periods for each location, with lockdown 1 (LD1), inter-lockdown 1 (LD1-LD2), lockdown 2 (LD2), and inter-lockdown 2 (LD2-LD3) covered by all six catchment areas, and lockdown 3 (LD3) and post-lockdown 3 (post-LD3) additionally covered by locations (d-f). The WWTP sampling period comprises LD3 and post-LD3 (Figure 2). Figure 2 shows per-site wastewater viral loads alongside reported case numbers. Administrated vaccination doses in Bavaria and reported COVID-19 deaths in Germany are shown in panels 2g and 2h [41], and overall COVID-19 deaths in Germany [42] in Figure 2g,h. Until September 2020, SARS-CoV-2 was only rarely detected, consistent with the very low case numbers of that period and with our parallel sero-epidemiological study [43]. Detection was similarly near-absent over the summer months of May–August 2021. Lockdown dates are listed in Table 3, Section 2.6.

After 1.5 years, three of the six initial locations were discontinued because they proved unsuitable or impractical. Location (b) Grosshadern collects WW from the LMU university hospital, where severely ill COVID-19 patients in intensive care did not contribute to the local sewage, on-site staff were screened daily and excluded when positive, and large volumes of uncontaminated process water from cleaning and clinical operations diluted the influent. Samples from this catchment were consistently low in SARS-CoV-2 and not representative of the resident population, while notification data assigned the hospital’s admitted patients to it, biasing the analysis. Locations (a) Langwieder Bach and (c) Savitstr. were not continued because sampling required road closures and incurred high personnel costs. Monitoring continued at (d) Gysslinger Becken, (e) Schenkendorfstr., and (f) Schmidbartlanger, where viral loads were frequently detectable, and sampling continued twice weekly.

### 3.2. Wastewater Viral Load Reflects Reported Case Numbers

**Figure A1:**
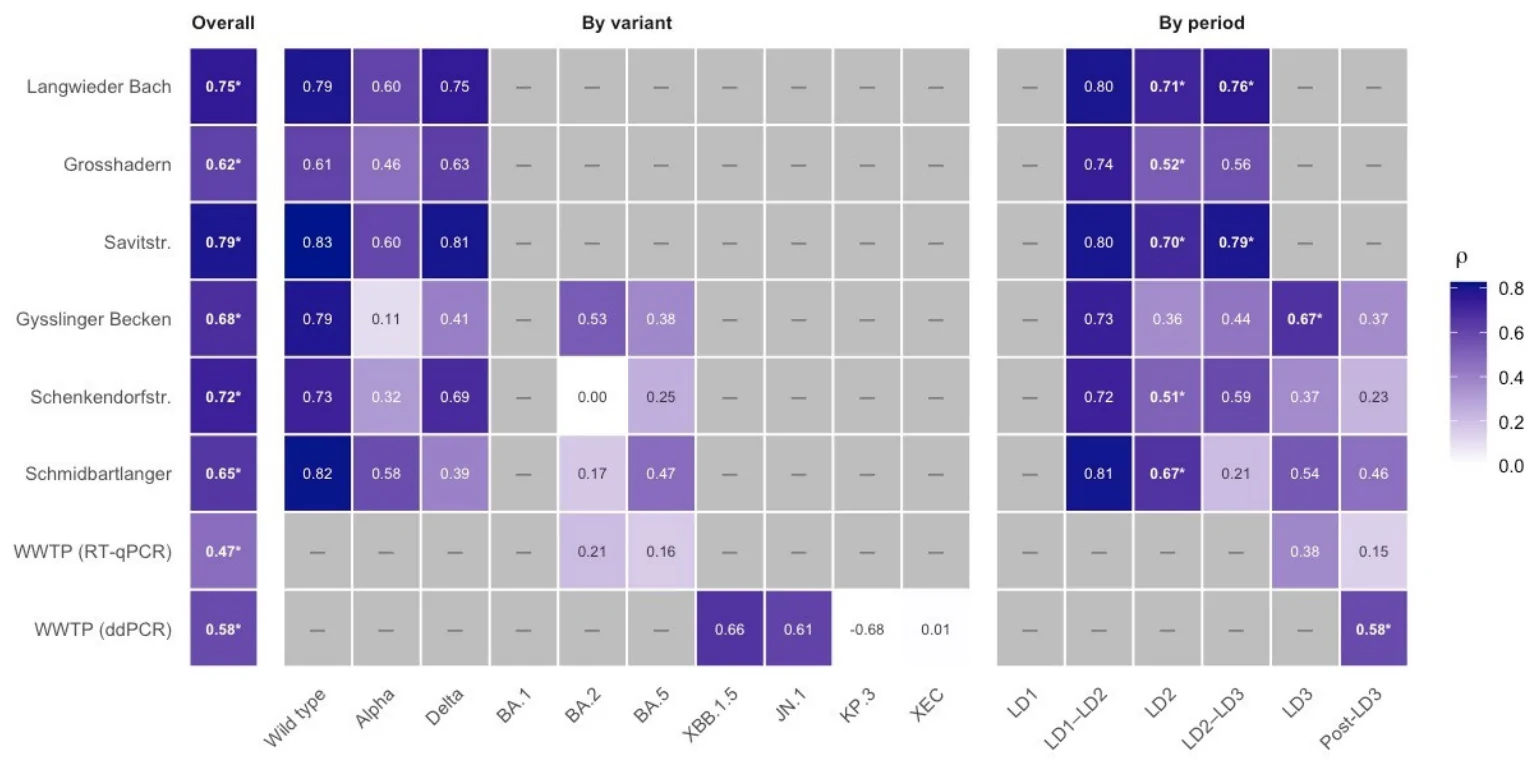
Correlation between wastewater SARS-CoV-2 viral loads and reported COVID-19 case numbers across sampling sites, variants, and pandemic periods in Munich. Spearman rank correlation coefficients (ρ) between weekly geometric-mean log_10_ viral loads and weekly log_10_ reported case counts are shown for each sampling site (rows), computed overall (**left**), stratified by dominant SARS-CoV-2 variant (**center**), and by predefined pandemic period (**right**). Cell color encodes ρ (see scale). Asterisks denote associations that remained significant after Bartlett and BH correction within each stratum. Gray cells (—) indicate strata with insufficient data for estimation. LD1–LD3: first to third lockdown. LD1–LD2 and LD2–LD3: the inter-lockdown intervals. Post-LD3: period following the third lockdown.

Weekly viral load tracked reported incidence consistently (Appendix A.1, Figure A1). Each site sampled site showed statistically significant positive level correlations overall (ρ ≈ 0.47–0.79) (Section 2.8). No week-over-week growth-rate correlation reached significance in any stratum. Level correlations tended to be stronger for the earlier variants (Wild-type, Alpha, and Delta) and periods (LD1-LD2, LD2, LD2-LD3), and weakened thereafter (Appendix A.1, Figure A1), likely reflecting reduced case reporting. Cross-correlation analysis revealed no consistent lead or lag at weekly resolution, with the optimal displacement at or near zero weeks.

Across each site’s full sampling period, Spearman correlations between wastewater SARS-CoV-2 viral loads and reported case numbers were higher for the residential catchments (ρ ≈ 0.62–0.79) than for the WWTP (ρ ≈ 0.47 and 0.58) (Appendix A.1, Figure A1). However, restricting the comparison to November 2021–November 2022, when the WWTP (RT-qPCR) and three of the residential catchments were sampled concurrently, left no systematic difference (residential ρ ≈ 0.44–0.58; WWTP ρ ≈ 0.47). The higher full-period correlations at residential sites therefore appear to reflect, at least in part, the greater clinical test coverage during their sampling periods. A residual site-type effect cannot be excluded and would be consistent with our previous report on influenza viruses in wastewater [44]. Decentralized sampling may resolve local SARS-CoV-2 infection dynamics more closely, because smaller catchments involve shorter in-sewer residence times, less sedimentation, and less decay within the sewer pipes. Confirming this advantage would require residential and treatment-plant sampling over a longer common period.

The overall level correlations are consistent with multi-site surveillance reported elsewhere. A nationwide U.S. analysis found a median across-state Spearman ρ of 0.6 (IQR 0.52–0.81) [45], and single-catchment studies report comparable coefficients that improve upon weekly aggregation [46]. The absence of a resolvable lead is expected, since reported lead times of wastewater over clinical cases are commonly only two to eight days [47]. The weakening of level correlations in the later periods is attributable to declining clinical test coverage rather than to failure of the wastewater signal. Reported case counts have been shown to diverge from wastewater concentrations once at-home antigen testing became widespread and PCR testing was restricted, with reported cases underestimated at the Omicron BA.2 peak [48,49]. Under effective sample size correction, all overall per-site level correlations remained significant (Appendix A.1, Table A1), whereas none of the by-variant and a minority of the by-period correlations did (Appendix A.1, Figure A1). With stratum-level significance tracking the number of effectively independent observations rather than the strength of the association, we interpret the stratified results by the magnitude and direction of the correlation coefficient rather than by significance.

### 3.3. Viral-Load-to-Case Ratio Under Changing Testing Conditions

To assess the robustness of WBE under changing surveillance conditions, we compared SARS-CoV-2 wastewater viral loads with reported case numbers as a log_10_ ratio over time, across all locations and the previously defined periods (Figure 3). For each location the ratio was referenced to a reliable-reporting baseline defined by its peak testing period, which is the interval during which most tests were conducted for the respective sampling period. These intervals for clinical data are mid-June 2021 to mid-June 2022 for locations (a-f), November 2021 to mid-June 2022 for the WWTP Gut Grosslappen measured by RT-qPCR, and the winter infection seasons of November 2022–February 2023 and October 2023–January 2024 for the WWTP Gut Grosslappen measured by ddPCR.

During phases of high testing capacity, the log_10_ ratio stayed close to its baseline, indicating that reported cases and wastewater signals tracked community circulation concordantly. After mandatory testing ended in March 2023, the ratio rose markedly (Figure 3h), reflecting a widening discrepancy in which wastewater continued to track viral circulation while reported cases were increasingly underestimated. This trend was modulated seasonally: during the colder autumn and winter months of 2023/24 and 2024/25, symptomatic individuals more often sought medical attention and were tested, so the ratio fell back toward baseline. It rose again through the following spring and summer as care-seeking and testing declined.

These patterns show that wastewater viral loads remained a more robust indicator of SARS-CoV-2 circulation even when clinical surveillance was incomplete. This provides a local indicator of possible case underascertainment, which in Section 3.2 could only be inferred indirectly from the weaker later-period correlations. Others have reported the same divergence once testing contracted [48,49].

### 3.4. Wastewater Dynamics During and After Lockdowns Differ

**Figure A2:**
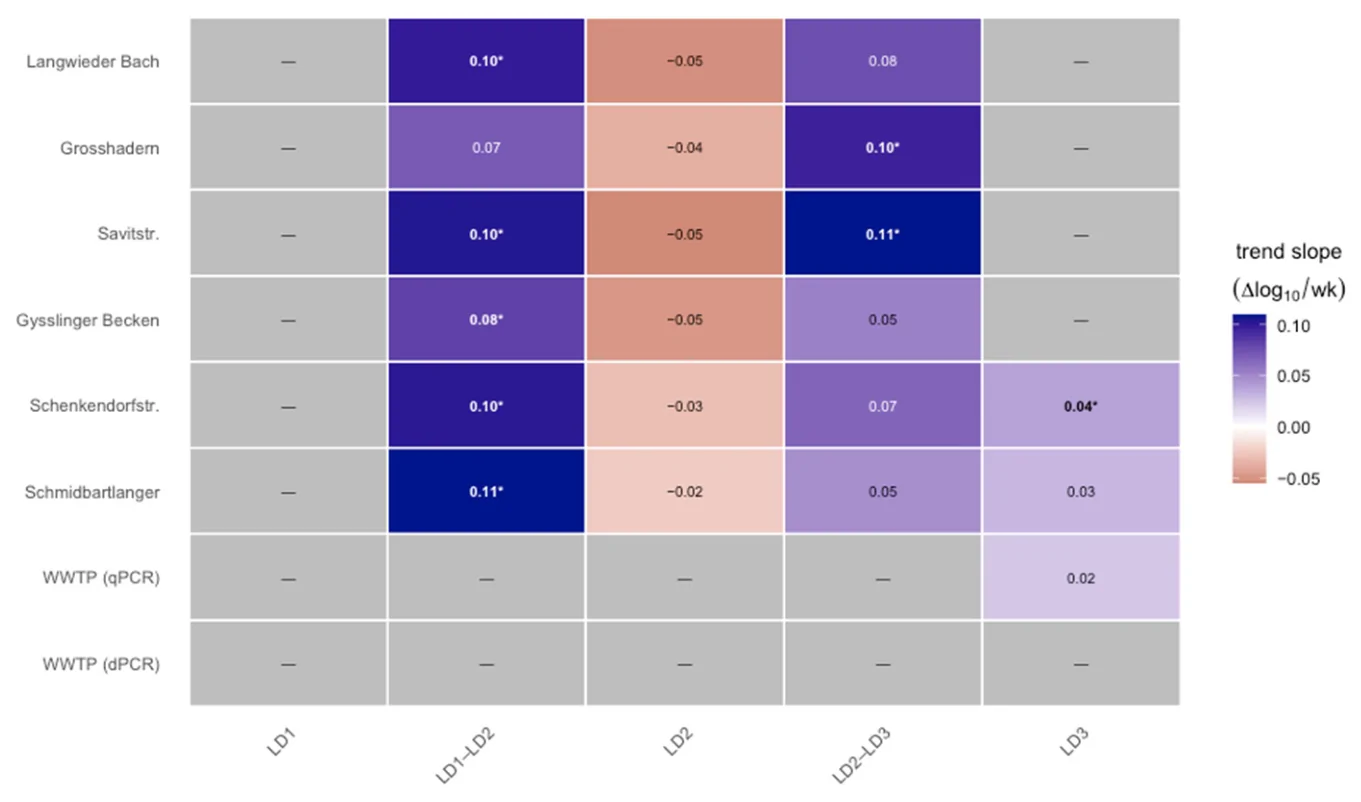
Wastewater SARS-CoV-2 trends and their relationship to lockdown stringency. Per-site (rows) and per-period (columns) trends in log_10_ viral load, expressed as slopes (Δlog_10_ per week) estimated by generalized least-squares regression with a continuous-time AR(1) error structure; cell color encodes slope direction and magnitude, asterisks denote significance after BH correction, and gray cells (—) indicate periods that were not estimable (n < 8 or non-convergence).

To assess the trends associated with lockdowns and lifting NPIs, viral load trends were quantified within each period and Spearman correlations were calculated between viral loads and the COVID-19 stringency index (Section 2.8). Results of the trend analysis are shown in Appendix A.2, Figure A2. During both inter-lockdown intervals, viral-load trends were increasing at all sites, with the positive slopes usually significant in the LD1–LD2 interval and significant at two of six sites during LD2–LD3. During LD2 itself, fitted slopes were slightly negative at every site but non-significant; thus, while clear evidence of a decline in concentrations during this lockdown is lacking, the strong positive growth seen before its start did not continue during LD2. For the less strict third lockdown, GLS models converged at three of the four sites with sufficient data, with (d) Gysslinger Becken failing to converge. All three slopes were slightly positive, with only (e) Schenkendorfstr. reaching significance (Appendix A.2, Table A2). Overall, restrictions coincided with periods of high infection activity and were relaxed as that activity receded. Because the timing of changes in transmission are confounded, the direction of influence cannot be determined from these data.

Meanwhile, higher stringency coincided with lower viral loads at the three most densely sampled sites (d-f) (Figure 4), with modest correlation coefficients of −0.30, −0.41, and −0.26 at Gysslinger Becken, Schenkendorfstr. and Schmidbartlanger, respectively, consistent with higher viral loads during periods of lower stringency. This result is consistent with early single-lockdown reports, in which stay-at-home orders were followed by clear declines in wastewater SARS-CoV-2 [47,50]. The stringency–load association is likely confounded by the coincidence of low stringency with the high-incidence Omicron waves from 2022 onward and should be read as correlational rather than causal. The same caution applies to formal non-pharmaceutical-intervention (NPI) analyses, in which strong temporal collinearity between successive measures makes causal attribution difficult even where transmission reductions are estimated [51]. After correction for autocorrelation, none of the apparent stringency-load correlations remained statistically significant (Appendix A.2, Table A3), and we describe these relationships only in terms of their magnitude and direction.

### 3.5. Variant-Specific Peaks in the Wastewater Signal

A consistent wild-type peak occurred in November 2020 at all six sites (Figure 5). One to two small Alpha-associated peaks appeared in March–April 2021 at sites (a–c), and a small Delta-associated peak was resolved at sites (a) and (e,f) in autumn 2021, with the exact month varying by site. Omicron BA.1 produced no clearly resolvable peak, consistent with its brief dominance before displacement by BA.2, whereas BA.2 generated a consistent peak in March 2022. A smaller but consistent BA.5-associated peak followed in late June–early July 2022, with a second peak at one site, (e) Schenkendorfstr., in September 2022. Droplet digital PCR (ddPCR) monitoring at the treatment-plant level extended coverage to later variants, resolving two XBB.1.5 peaks in February and October 2023, no distinct JN.1 peak, a small KP.3 peak in July 2024, and a small XEC peak at the very end of 2024.

The timing of these peaks corresponds closely to the local variant-succession timeline, indicating that the wastewater signal registered each wave within the interval over which the incoming variant reached local dominance. This alignment is corroborated by independent German wastewater surveillance, where BA.4/BA.5-specific RNA was detected in wastewater in another Federal State from May 2022 and formed the majority fraction by June 2022, ahead of BA.4/BA.5 becoming nationally dominant mid-July 2022 [52]. This is consistent with the BA.5-associated peak resolved here in June–July 2022. The failure to resolve a BA.1 peak reflects its rapid replacement by BA.2. However, a sensitivity analysis of LOESS span and prominence threshold revealed that some of the smaller, site-variable Alpha, Delta, and Omicron BA.5 peaks were more sensitive to these parameters and should be interpreted with caution, while the major wild-type and BA.2 peaks were consistently recovered (Appendix A.3, Table A4). Peak timing was highly stable and additional peaks were largely absent except under the most under-smoothed configuration.

### 3.6. Wastewater Viral Load Versus Google Trends Search Interest

Comparison of Google Trends data with wastewater viral loads (Figure 6) showed a clear association only during the first inter-lockdown interval, where four of six sites correlated strongly (ρ ≈ 0.8) with viral load leading search interest by zero to three days; the association attenuated at all sites in every following period. This is consistent with the established behavior of search-based indicators, where the signal is dominated by public attention and media coverage rather than infection dynamics [40,53]. It indicates that search interest is not a reliable complement to wastewater surveillance once a pandemic becomes routine.

### 3.7. Mass Gatherings and Wastewater SARS-CoV-2 Dynamics

During the first two pandemic years, most large events were cancelled to reduce transmission. From 2022 they resumed, initially under regulations and later without. Fixed-period, longer mass gatherings such as Christmas markets and the *Oktoberfest* coincided with periods of rising case numbers and wastewater viral loads. Christmas markets fall within the usual respiratory-infection season, so any contribution they may have made cannot easily be separated from ordinary seasonal changes.

The *Oktoberfest* returned in autumn 2022 after its 2020–2021 suspension, drawing millions of visitors. Between mid-September and mid-October 2022, wastewater viral loads for SARS-CoV-2 rose across the WWTP and residential catchments (d–f), peaking in early October (Figure 2). A comparable September–November rise, mirrored by reported cases, was observed in 2023 and 2024. In 2021, when the event did not take place, viral loads remained low through September, with the autumn rise beginning in mid-October and peaking in early November (Figure 2). We report this correspondence in timing as a descriptive observation only, as the present data do not support an estimate of the event’s contribution to wave onset, and no causal inference is drawn. A formal analysis of autumn wave-onset timing in relation to mass gatherings is the subject of a separate study.

### 3.8. Limitations of the Study

Several methodological limitations should be considered when interpreting the findings of this longitudinal study. First, at the study’s inception in April 2020, standard operational protocols for wastewater sampling and RT-qPCR assays for SARS-CoV-2 were not yet fully established, requiring iterative optimization that may introduce minor baseline variability.

Second, systematic biomarker normalization utilizing Pepper Mild Mottle Virus (PMMoV) was only fully integrated into the workflow in November 2022. Consequently, some samples collected prior to this date were normalized retrospectively using nucleic acid eluates that had undergone a single additional freeze–thaw cycle from deep-frozen storage. Although prior internal validations indicate that an extra freeze–thaw cycle does not significantly alter cycle threshold (C_t_) values, the potential for minor RNA degradation cannot be entirely discounted. Furthermore, PMMoV normalization relies on constant per-capita pepper consumption, an assumption that has not been verified for the Bavarian population and that may be affected by seasonality or long-term dietary shifts. While systematic variations in PMMoV shedding would directly affect normalized viral loads, concentrations in our five-year dataset remained within the range of 6–7 log_10_ units, indicating that major marker drift was unlikely.

**Figure A3:**
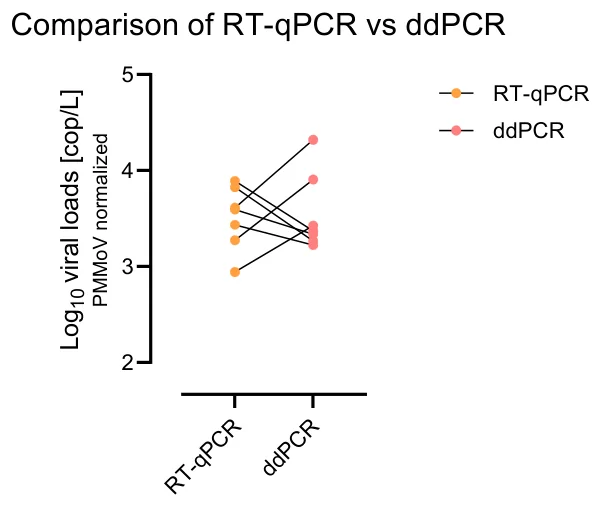
Cross-method validation of RT-qPCR and droplet digital PCR (ddPCR) for wastewater-based epidemiology. Individual data points represent the log_10_ viral loads (cop/L) of SARS-CoV-2 normalized against Pepper Mild Mottle Virus (PMMoV) internal controls. Solid black lines directly connect split eluates analyzed in parallel by both platforms to illustrate sample-specific tracking. While the majority of samples cluster consistently between 3.2 and 3.9 log_10_ cop/L across both methods, specific temporal samples exhibit a marked increase in absolute quantification when utilizing the higher-sensitivity ddPCR assay.

Finally, the quantification methodology for the WWTP was transitioned from RT-qPCR to ddPCR 2.5 years into the study, when the catchment samples were discontinued. While ddPCR offers superior analytical sensitivity and enables the quantification of ultra-low viral copy numbers, it also showed differences in absolute quantification compared to RT-qPCR. Paired RT-qPCR and ddPCR measurements agreed within the same order of magnitude, but the direction of divergence was inconsistent across the seven paired samples (median ddPCR/RT-qPCR ratio 0.62, Appendix A.4, Table A5). Because these two methodologies capture distinct molecular dynamics and were deployed sequentially rather than concurrently over the entire study duration, direct cross-method comparisons of absolute maximum viral loads remain constrained (Appendix A.4, Figure A3, Table A5). Cross-validation requires an extended period and greater sampling size of parallel side-by-side quantification to establish an accurate mathematical conversion factor.

## 4. Conclusions

Wastewater-based epidemiology (WBE) of SARS-CoV-2 was established at the start of the COVID-19 pandemic, before it was clear that the approach would work for this virus. Our study spans five years, from April 2020 to April 2025, covering several residential catchment areas in Munich and, later, the WWTP Gut Grosslappen, across lockdown, inter-lockdown, and post-lockdown phases. By comparing SARS-CoV-2 wastewater viral loads with officially reported clinical data for Munich and the corresponding residential areas, we provide a longitudinal, spatio-temporal record of SARS-CoV-2 circulation in the city.

The infection waves resolved in wastewater and patient swab data moved in parallel, indicating that wastewater viral loads reflect population-level infection dynamics. A decline in viral loads was observed during the second lockdown but not the third. Because restrictions were introduced in response to rising infection numbers, changes in stringency and changes in transmission are confounded, so these observations remain correlational and do not permit conclusions about the direction of influence. Switching from RT-qPCR to ddPCR improved detection of lower viral loads, particularly during the summer months when infection activity was low. Notably, the ratio of wastewater viral loads to reported cases showed that, once mandatory testing was suspended, notification numbers no longer reflected actual case numbers, indicating underascertainment, while wastewater continued to track viral circulation.

This study shows that the epidemiological value of wastewater surveillance depends on the intensity of clinical testing. While mandatory testing was in place, wastewater viral loads and reported cases were highly concordant. As testing contracted, the two diverged: wastewater continued to track infection dynamics while reported cases increasingly underestimated them. This pattern can only be observed in a record spanning both surveillance regimes.

Together, these findings demonstrate that wastewater data are a reliable tool for monitoring SARS-CoV-2 in a population. WBE offers a cost-effective, timely, and anonymous means of population-level surveillance that does not depend on individual test-seeking, clinical personnel, or medical appointments, and it remains informative precisely when clinical surveillance breaks down.

## Figures and Tables

**Table 1:** Sampling site characteristics, time periods, and number of analyzed samples.

Location of Sampler	Permanent Inhabitants	Drainage Size [ha]	Time Period of Sampling	Samples Analyzed in Total [n]
(a) Langwieder Bach	9500	200	04/2020–11/2021	73
(b) Grosshadern	6700	85	04/2020–11/2021	73
(c) Savitstr.	145,500	2300	04/2020–11/2021	73
(d) Gysslinger Becken	157,900	1150	04/2020–11/2022	177
(e) Schenkendorfstr.	118,300	1050	04/2020–11/2022	175
(f) Schmidbartlanger	66,990	670	04/2020–11/2022	174
(g) WWTP Gut Grosslappen	961,000	20,000	11/2021–04/2025	355

**Table 3:** Information on lockdown time periods in the Federal State of Bavaria.

Lockdown Status	Start	End
Lockdown 1 (LD1)	16 March 2020	16 June 2020
Inter-lockdown 1 (LD1-LD2)	17 June 2020	8 December 2020
Lockdown 2 (LD2)	9 December 2020	6 June 2021
Inter-lockdown 2 (LD2-LD3)	7 June 2021	10 November 2021
Lockdown 3 (LD3)	11 November 2021	11 May 2022
Post-lockdown 3	12 May 2022 onwards	-

## Data Availability

Data on reported case numbers per sewer catchment area, using zip codes, were obtained from the Munich health authority (GSR) and are available upon request. Data on Google Trends are openly available at https://trends.google.de/trends/ (accessed on 6 August 2026). The original contributions presented in this study are included in the article. Upon request the analysis details and codes can be shared. Further inquiries can be directed to the corresponding author.

## Abbreviations

The following abbreviations are used in this manuscript:

24h	Twenty-four hour
BayVOC	Molecular surveillance of infectious pathogens in Bavaria
BH	Benjamini–Hochbeg
CDC	Centers for disease control and prevention
cop/L	Copies per liter
corCAR1	Continuous-time first-order autoregression
COVID-19	Coronavirus disease of 2019
ddPCR	Droplet digital polymerase chain reaction
GLS	Generalized least-square
GSR	Munich Health Authority (Gesundheitsreferat München)
IfSG	German Infection Protection Act (Infektionsschutzgesetz)
IQR	Inter-quartile range
LD	Lockdown
LOESS	Locally Estimated Scatterplot
LOWESS	Locally weighted scatterplot smoothing
MGE	Mass-gathering-event
MSE	Munich Sewer Authority (Münchner Stadtentwässerung)
n	number
ORF	Open reading frame
OxCGRT	Oxford COVID-19 government response tracker
PCR	Polymerase chain reaction
PMMoV	Pepper mild mottle virus
RNA	Ribonucleic acid
RT-qPCR	Reverse transcription quantitative polymerase chain reaction
SARS-CoV-2	Severe acute respiratory syndrome coronavirus 2
US	United States
WBE	Wastewater-based epidemiology
WBS	Wastewater-based surveillance
WW	Wastewater
WWTP	Wastewater treatment plant
µL	Microliter

## Appendix A

### Appendix A.1. Spearman Correlation of Wastewater SARS-CoV-2 Viral Loads and Reported Cases

**Table A1:** Adjusted *p*-values per site for correlation of viral loads of SARS-CoV-2 in wastewater and respective weekly data points and Bartlett-corrected effective sample sizes.

Catchment Area	Adjusted *p*-Value	Weekly Data Points [n]	Effective Weekly Data Points [n]
(a) Langwieder Bach	4.86 × 10^−4^	73	22
(b) Grosshadern	6.60 × 10^−4^	73	30
(c) Savitstr.	5.02 × 10^−4^	73	17
(d) Gysslinger Becken	2.77 × 10^−3^	124	18
(e) Schenkendorfstr.	3.80 × 10^−3^	124	14
(f) Schmidbartlanger	2.77 × 10^−3^	124	20
WWTP Gut Grosslappen (RT-qPCR)	7.40 × 10^−3^	51	32
WWTP Gut Grosslappen (ddPCR)	7.40 × 10^−3^	126	20

### Appendix A.2. Wastewater SARS-CoV-2 Trends and Their Relationship to Lockdown Stringency

**Table A2:** Adjusted *p*-values per site for trend analysis per lockdown period of viral loads of SARS-CoV-2 in wastewater.

Catchment Area	*p*-Value LD1	*p*-Value LD 1-LD2	*p*-Value LD2	*p*-Value LD 2-LD3	*p*-Value LD3
(a) Langwieder Bach	-	3.54 × 10^−5^	1.49 × 10^−1^	7.73 × 10^−2^	-
(b) Grosshadern	-	1.74 × 10^−1^	1.59 × 10^−1^	1.45 × 10^−2^	-
(c) Savitstr.	-	2.48 × 10^−4^	2.25 × 10^−1^	4.26 × 10^−2^	-
(d) Gysslinger Becken	-	3.51 × 10^−3^	1.59 × 10^−1^	1.59 × 10^−1^	-
(e) Schenkendorfstr.	-	3.54 × 10^−5^	2.40 × 10^−1^	8.65 × 10^−2^	4.26 × 10^−2^
(f) Schmidbartlanger	-	6.27 × 10^−4^	1.59 × 10^−1^	7.73 × 10^−2^	1.33 × 10^−1^
WWTP Gut Grosslappen (RT-qPCR)	-	-	-	-	2.06 × 10^−1^

**Table A3:** Spearman correlation coefficients, adjusted *p*-values, and Bartlett-corrected effective sample sizes for the stringency correlation analysis of viral loads of SARS-CoV-2 in wastewater.

Catchment Area	Correlation Coefficient	Adjusted *p*-Value	Effective Sample Size [n]
(a) Langwieder Bach	−0.07	0.94	18
(b) Grosshadern	−0.06	0.94	21
(c) Savitstr.	0.00	1.00	15
(d) Gysslinger Becken	−0.30	0.50	19
(e) Schenkendorfstr.	−0.41	0.50	15
(f) Schmidbartlanger	−0.26	0.50	22
WWTP Gut Grosslappen (RT-qPCR)	0.20	0.50	31

### Appendix A.3. Variant-Specific Peaks in Wastewater Concentration Trajectories

**Table A4:** Detection rates and peak-date ranges for the sensitivity analysis of variant-specific wastewater peaks across 16 combinations of LOESS span (0.15, 0.20, 0.25, 0.30) and prominence threshold (0.20, 0.30, 0.40, 0.50).

Catchment Area	Variant	Peak Date (Main Analysis)	Prop. of Settings Detecting Peak	Peak-Date Range (Weeks)
(a) Langwieder Bach	Wild type	30 November 2020	1.00	1
(a) Langwieder Bach	Alpha	5 April 2021	1.00	0
(a) Langwieder Bach	Delta	30 August 2021	0.38	0
(b) Grosshadern	Wild type	23 November 2020	1.00	2
(b) Grosshadern	Alpha	1 March 2021	0.44	1
(b) Grosshadern	Alpha	19 April 2021	0.38	0
(c) Savitstr.	Wild type	16 November 2020	1.00	1
(c) Savitstr.	Alpha	19 April 2021	1.00	1
(d) Gysslinger Becken	Wild type	30 November 2020	1.00	1
(d) Gysslinger Becken	Omicron BA.2	14 March 2022	1.00	1
(d) Gysslinger Becken	Omicron BA.5	20 June 2022	0.56	1
(e) Schenkendorfstr.	Wild type	9 November 2020	1.00	2
(e) Schenkendorfstr.	Delta	22 November 2021	0.56	1
(e) Schenkendorfstr.	Omicron BA.2	28 March 2022	1.00	1
(e) Schenkendorfstr.	Omicron BA.5	20 June 2022	0.38	0
(e) Schenkendorfstr.	Omicron BA.5	5 September 2022	0.56	2
(f) Schmidbartlanger	Wild type	16 November 2020	0.69	2
(f) Schmidbartlanger	Delta	6 September 2021	0.94	3
(f) Schmidbartlanger	Omicron BA.2	14 March 2022	1.00	3
(f) Schmidbartlanger	Omicron BA.5	4 July 2022	0.63	2
WWTP Gut Grosslappen (RT-qPCR)	Omicron BA.2	21 March 2022	1.00	1
WWTP Gut Grosslappen (RT-qPCR)	Omicron BA.5	27 June 2022	0.88	1
WWTP Gut Grosslappen (ddPCR)	Omicron XBB.1.5	20 February 2023	1.00	1
WWTP Gut Grosslappen (ddPCR)	Omicron XBB.1.5	2 October 2023	1.00	1
WWTP Gut Grosslappen (ddPCR)	Omicron KP.3	1 July 2024	1.00	3
WWTP Gut Grosslappen (ddPCR)	Omicron XEC	30 December 2024	0.75	3

### Appendix A.4. Methodological Comparison of SARS-CoV-2 Quantification with RT-qPCR and ddPCR in Wastewater

**Table A5:** Measured values of cross-method validation of RT-qPCR and ddPCR quantification of SARS-CoV-2 in wastewater.

Date of Collection	RT-qPCR [cop/L]	ddPCR [cop/L]	Ratio (ddPCR/RT-qPCR)
27 September 2022	2714.75	1670.00	0.62
30 September 2022	4108.11	20,904.09	5.09 *
7 October 2022	7807.44	2344.68	0.30
21 October 2022	6707.18	1858.33	0.28
25 October 2022	874.91	2670.11	3.05
27 October 2022	3914.29	2175.04	0.56
4 November 2022	1882.81	8064.15	4.28
Median	3914.29	2344.68	0.62
Range	874.91–7807.44	1670.00–20,904.09	0.28–5.09
Mean	4001.36	5669.49	-

* This sample showed the largest divergence between the analysis platforms. No technical cause could be determined. The pair was retained in dataset.

### Appendix A.5. Higher Infection Dynamics in 2022 than in 2021

Viral loads in wastewater (WW) and numbers of reported case numbers were compared between years 2021 and 2022. The year 2022 marked elevated numbers for both variables. Table A6 displays median viral loads and case numbers for each year.

**Table A6:** Median viral loads (samples above LOD) and reported case numbers to the health authority Munich (GSR) for locations (d-f) in 2021 and 2022.

Catchment Area	Viral Loads WW 2021 [cop/L]	Viral Loads WW 2022 [cop/L]	Weekly Reported Case Numbers 2021 [n]	Weekly Reported Case Numbers 2022 [n]
(d) Gysslinger Becken	1216	6131	121	1016.5
(e) Schenkendorfstr.	2253	5904	78	755
(f) Schmidbartlanger	3730	6661	104	313.5
